# The Assessment of Post-Vasectomy Pain in Mice Using Behaviour and the Mouse Grimace Scale

**DOI:** 10.1371/journal.pone.0035656

**Published:** 2012-04-25

**Authors:** Matthew C. Leach, Kristel Klaus, Amy L. Miller, Maud Scotto di Perrotolo, Susana G. Sotocinal, Paul A. Flecknell

**Affiliations:** 1 Institute of Neuroscience and Comparative Biology Centre, Newcastle University, Newcastle upon Tyne, United Kingdom; 2 Department of Psychology and Alan Edwards Centre for Research on Pain, McGill University, Montreal, Quebec, Canada; Baylor College of Medicine, Jiao Tong University School of Medicine, United States of America

## Abstract

**Background:**

Current behaviour-based pain assessments for laboratory rodents have significant limitations. Assessment of facial expression changes, as a novel means of pain scoring, may overcome some of these limitations. The Mouse Grimace Scale appears to offer a means of assessing post-operative pain in mice that is as effective as manual behavioural-based scoring, without the limitations of such schemes. Effective assessment of post-operative pain is not only critical for animal welfare, but also the validity of science using animal models.

**Methodology/Principal Findings:**

This study compared changes in behaviour assessed using both an automated system (“HomeCageScan”) and using manual analysis with changes in facial expressions assessed using the Mouse Grimace Scale (MGS). Mice (n = 6/group) were assessed before and after surgery (scrotal approach vasectomy) and either received saline, meloxicam or bupivacaine. Both the MGS and manual scoring of pain behaviours identified clear differences between the pre and post surgery periods and between those animals receiving analgesia (20 mg/kg meloxicam or 5 mg/kg bupivacaine) or saline post-operatively. Both of these assessments were highly correlated with those showing high MGS scores also exhibiting high frequencies of pain behaviours. Automated behavioural analysis in contrast was only able to detect differences between the pre and post surgery periods.

**Conclusions:**

In conclusion, both the Mouse Grimace Scale and manual scoring of pain behaviours are assessing the presence of post-surgical pain, whereas automated behavioural analysis could be detecting surgical stress and/or post-surgical pain. This study suggests that the Mouse Grimace Scale could prove to be a quick and easy means of assessing post-surgical pain, and the efficacy of analgesic treatment in mice that overcomes some of the limitations of behaviour-based assessment schemes.

## Introduction

Legislation governing the use of animal in biomedical research requires that any unnecessary pain or distress is avoided or alleviated (e.g. European Directive EU 2010/63). Successful implementation of effective pain management strategies in animals requires accurate assessment of post-surgical/procedural pain. Such assessments are also essential for evaluating animal models used in the development of novel analgesics. Behaviour-based assessments of pain have been developed for both rats and mice following surgery and other traumatic procedures, and use either the appearance of abnormal behaviours [1]–[4], or the change in the frequency of normal behaviour patterns [5] to score pain. The latter approach has the advantage of enabling automated as well as manual behavioural assessments to be conducted, and has been recommended in expert reports [6]. Despite the obvious advantages of using behaviour to assess pain in animals, there remain a number of limitations. The non-specific (i.e. non-analgesic) effects of many commonly used opioids (e.g. buprenorphine, morphine) can confound behavioural assessments by causing marked behavioural changes in normal, pain-free rodents (e.g. altered activity, increased grooming etc.) that can overlap with those considered to be associated with pain [7]. These changes in overall activity levels could also influence the exhibition abnormal behaviours, so extending this problem to both types of behavioural assessment.

The specific behavioural responses to painful stimuli may also vary markedly following different surgical or other painful procedures. Currently such behaviours have been identified for a very limited range of procedures in a small number of laboratory animal species, e.g. abdominal-based procedures in rats, mice and rabbits [2]–[4]. A more fundamental issue relates to the underlying assumption that behavioural responses reflect an animal's integrated response to external stimuli and relate directly to its internal state. However, they may simply reflect the response to the sensory afferent barrage associated with tissue damage (nociceptive input), and not reflect the affective component of pain (‘how pain makes animals feel’) [8], [9]. It is this affective component that is most relevant from a welfare perspective (as recognised in humans).

The recently described approach of using facial expressions to assess pain [9] may overcome many of these difficulties. The authors demonstrate that mice undergoing routine rodent nociceptive tests exhibit characteristic changes in facial expressions. Based on these expressions the authors have developed the Mouse Grimace Scale (MGS), which has been used to score pain intensity [9]. In this study, morphine administration induced no change in facial expressions in normal (pain-free) laboratory mice, suggesting no confounding influence of opioid analgesia. Preliminary data from Langford et al. [9] also raises the possibility that facial expression could indicate the affective component of pain in animals as it does in humans. Lesioning of the rostral anterior insula (implicated in the affective component of pain in humans) prevented changes in facial expression but not abdominal writhing (the behavioural marker of abdominal pain or nociception). In addition, using facial expressions to assess pain should be less time consuming to apply than full behavioural scoring, allowing effective indicators of pain to be rapidly identified for a greater range of procedures. All of the indicators are located in one small area (i.e. face), so exploiting the human tendency to focus on animal faces when assessing pain [10]. Analysing facial expressions may also offer increased sensitivity, as Langford et al. [9] have determined dose response curves for a range of painful stimuli and analgesia, which has yet to be done with behavioural analysis.

This study compares manual and automated behavioural analysis with the Mouse Grimace Score (MGS) for assessing post-vasectomy pain in mice. Vasectomy is carried out as a routine procedure in most facilities that produce genetically altered (transgenic) mice. It was considered a suitable procedure for assessment of the MGS, as the behavioural effects of the procedure had been investigated [4] and an on-going requirement for the procedure in our facility avoided undertaking surgical procedures solely for the evaluation of post-procedural pain. A further goal of the study was to indicate whether the MGS could be successfully implemented with minimal training, enabling an effective “cage-side” assessment to be developed.

## Results

### Mouse Grimace Scale (MGS)

**Figure 1:**
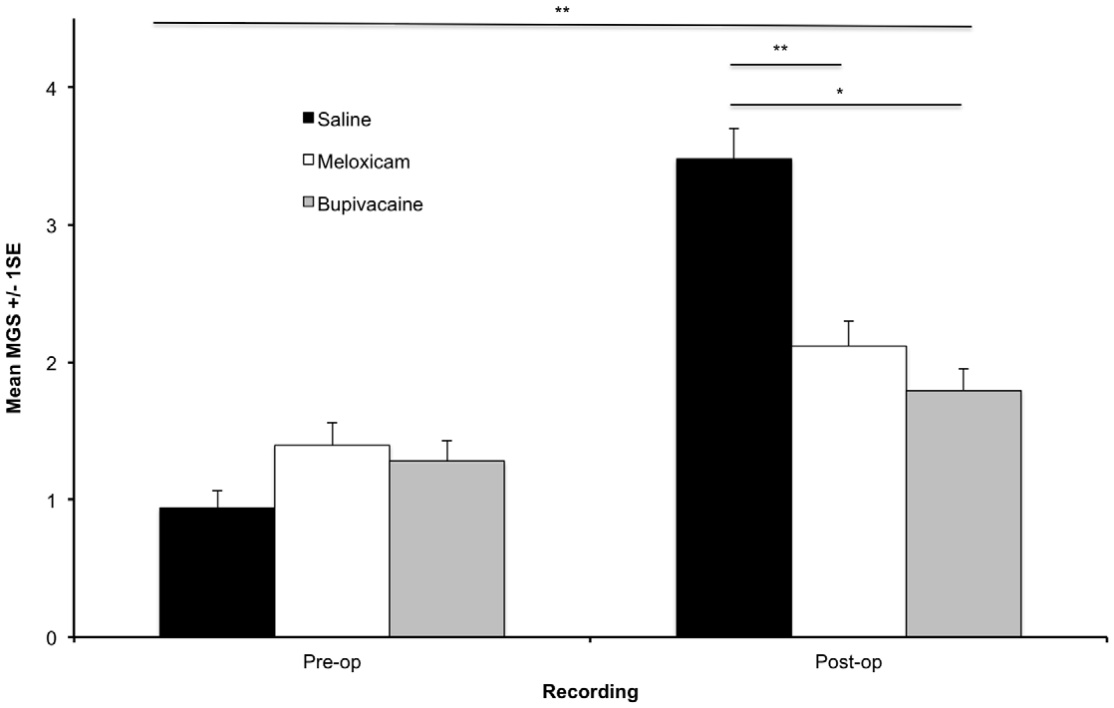
Mean Mouse Grimace Scale scores pre and post vasectomy. MGS scores are presented on the y-axis (± 1SE) for mice receiving 2 ml/kg Saline, 20 mg/kg Meloxicam and 5 mg/kg Bupivacaine with the pre and post vasectomy recordings on the x-axis (^★^P = 0.002, ^★★^P = 0.000).

Time, treatment and a time*treatment interaction had significant effects on the MGS (P = 0.000, P = 0.000, P = 0.007 respectively). The MGS score was significantly higher post compared to pre-operatively (P = 0.000). There was no significant difference between the treatments in the pre-operative period (P = 0.11). Post-operatively the MGS score was significantly higher in the saline compared to Meloxicam and Bupivicaine treated groups (P = 0.000, P = 0.002 respectively), with no difference between the Meloxicam and Bupivicaine treated groups (P = 0.69) (see Figure 1).

### Manual behaviour analysis

#### Pain behaviour

**Figure 2:**
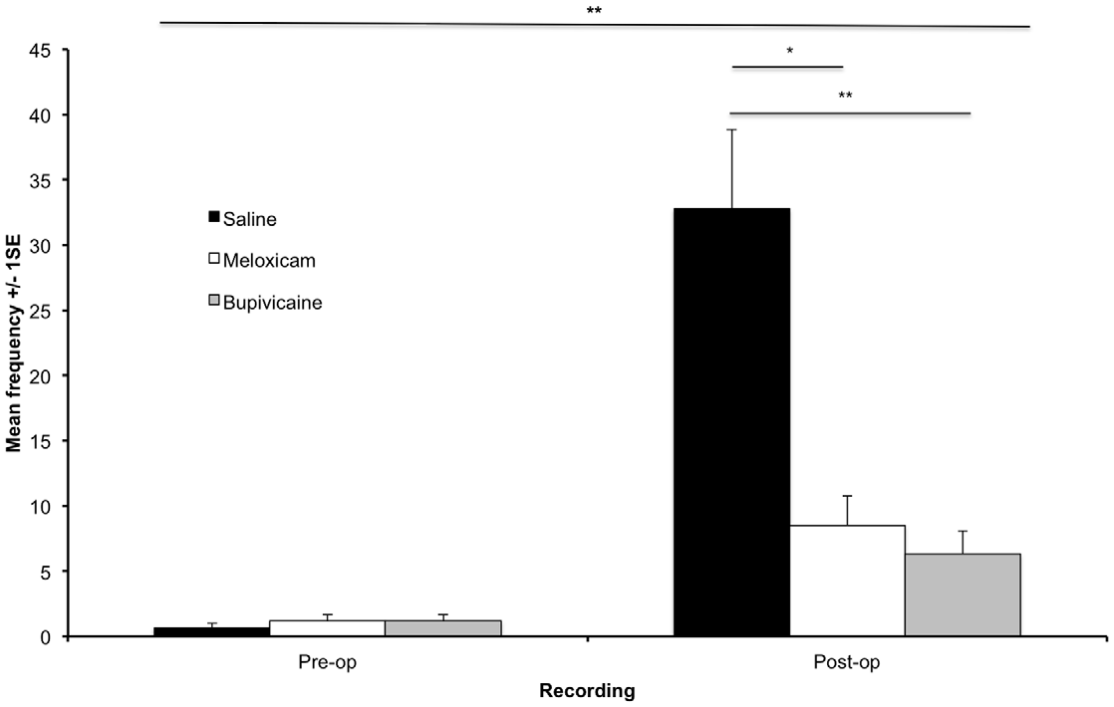
Mean frequency of the composite pain scores pre and post vasectomy. Composite pain scores are presented on the y-axis (± 1SE) for mice receiving 2 ml/kg Saline, 20 mg/kg Meloxicam and 5 mg/kg Bupivacaine with the pre and post vasectomy recordings on the x-axis (^★^P = 0.001, ^★★^P = 0.000).

Many of the individual pain-related behaviours were observed too infrequently to be meaningful, so those showing the same pattern were amalgamated to form a composite pain score, comprising; arch, circle, fall, flinch, press, rear leg lift, stagger, twitch and writhe. Time, treatment and a time*treatment interaction had significant effects on the frequency of pain behaviour (P = 0.000 for all comparisons). The frequency of pain behaviour was significantly greater in the postoperative compared to pre-operative period (P = 0.000). There was no significant difference between the treatment groups pre-operatively (P = 0.40), but there was post-operatively (P = 0.000), the frequency of pain behaviour was significantly greater in the saline treated animals compared to the Meloxicam and Bupivacaine treated animals (P = 0.001, P = 0.000 respectively), with no difference between the Meloxicam and Bupivacaine treated groups (P = 0.99) (see Figure 2).

#### General Grooming

Time had a significant effect on the frequency and duration of grooming (P = 0.001, P = 0.005 respectively), with the frequency and duration of grooming being lower pre compared to post-operatively. There was no effect of treatment (P = 0.20, P = 0.27 respectively) or time*treatment interaction (P = 0.10, P = 0.42 respectively) on grooming.

#### Wound Lick

Time had a significant effect on the frequency and duration of wound licking (P = 0.000 for both comparisons). Wound licking was only observed during the post-operative observations. There was no effect of treatment (P = 0.09, P = 0.30 respectively) or time*treatment interaction (P = 0.09, P = 0.30 respectively) on wound licking.

#### Rear

Time had a significant effect on the frequency and duration of rearing (P = 0.000 for both comparisons), with the frequency and duration of rearing was higher pre compared to post-operatively. There was no effect of treatment (P = 0.43, P = 0.32 respectively) or time*treatment interaction (P = 0.38, P = 0.45 respectively) on rearing.

### Automated behaviour analysis

A number of the behaviours scored by HomeCageScan decreased in frequency from the pre to post-operative period (see Table 1). A number of the behaviours scored by HomeCageScan increased in frequency from the pre to post-operative period: groom (P = 0.000), pause (P = 0.008) and remain partial rear (P = 0.004). However treatment or time*treatment interaction had no significant effect on the frequency of these behaviours post-operatively (see Table 2).

**Table 1:** The HomeCageScan scored behaviours that significantly decreased in frequency from pre to post vasectomy.

Behaviour (P-value)	
Come down (P = 0.000)	Jump (P = 0.000)
Rear up (P = 0.000)	Come down to partial rear (P = 0.001)
Remain rear up (P = 0.000)	Rear up from partial rear (P = 0.003)
Stretch (P = 0.002)	Unknown (P = 0.008)
Land vertically (P = 0.000)	Remain hang vertically (P = 0.01)
Walk left (P = 0.000)	Hang vertically from rear up (P = 0.000)
Walk right (P = 0.000)	Turn (P = 0.048)
Walk slow (P = 0.002)	Rear up to partial rear (P = 0.052)

**Table 2:** The HomeCageScan scored behaviours that were unaffected by treatment or time*treatment interaction post vasectomy.

Behaviour (P-value: treatment, time*treatment interaction)	
Come down (P = 0.84, P = 0.88)	Come down to partial rear (P = 0.19, P = 0.56)
Rear up (P = 0.77, P = 0.53)	Rear up from partial rear (P = 0.16, P = 0.93)
Remain rear up (P = 0.33, P = 0.09)	Remain partial rear (P = 0.45, P = 0.19)
Stretch (P = 0.61, P = 0.87)	Unknown (P = 0.69, P = 0.69)
Land vertically (P = 0.08, P = 0.08)	Remain hang vertically (P = 0.12, P = 0.09)
Walk left (P = 0.37, P = 0.50)	Turn (P = 0.90, P = 0.58)
Walk right (P = 0.55, P = 0.83)	Rear up to partial rear (P = 0.73, P = 0.93)
Walk slow (P = 0.47, P = 0.47)	Groom (P = 0.2, P = 0.58)
Jump (P = 0.74, P = 0.42)	Hang vertically from rear up (P = 0.07, P = 0.08)
Pause (P = 0.57, P = 0.45)	

#### Remain Low

Time*Treatment had a significant effect on the frequency of remain low (P = 0.038). There was no difference between the treatments post-operatively, but in the pre-operative period saline treated animals showed a higher frequency of remain low than meloxicam treated animals. Time or treatment alone had so significant effect on remain low (P = 0.78, P = 0.31 respectively).

#### Behaviours showing no change

Time, treatment or time*treatment interaction had no significant effect on hang cuddled (P = 0.97, P = 0.89, P = 0.82), come down from partial rear (P = 0.25, P = 0.93, P = 0.61), stationary (P = 0.69, P = 0.29, P = 0.32), hang vertically from hang cuddled (P = 0.51, P = 0.50, P = 0.72), repeated jumping (P = 0.08, P = 0.71, P = 0.62), sniff P = 0.08, P = 0.45, P = 0.17) and remain hang cuddled (P = 0.16, P = 0.84, P = 0.27).

### Relationship between behaviour and MGS

#### Manual behaviour scoring

**Figure 3:**
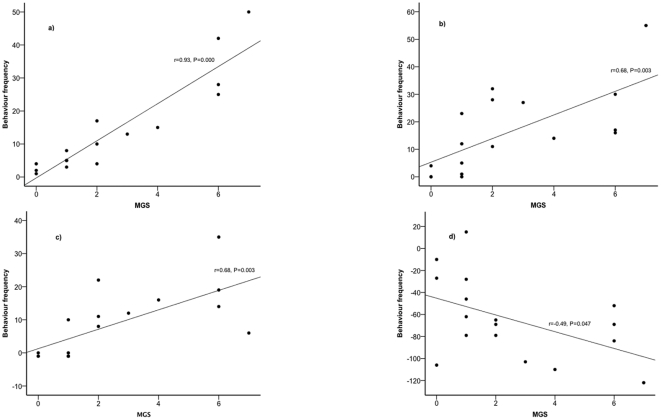
Relationship between changes in MGS and manually scored behaviour. MGS scores of one experienced observer are presented on the x-axis and manually scored behaviours from pre to post vasectomy are presented on the y-axis; composite pain behaviour (a), wound lick (b), grooming (c) and rearing (d).

The change in MGS from the pre to post-operative period was correlated with the change in a number of manually scored behaviours (see Figures 3a, b, c, d). The change in MGS score was positively correlated with the frequency of pain behaviours (r = 0.93, P = 0.000), wound lick (r = 0.68, P = 0.003) and groom (r = 0.68, P = 0.003). An increase in the MGS score from the pre to post-operative period was associated with an increase in the frequency of these behaviours. The change in MGS was negatively correlated with the frequency of rearing (r = −0.49, P = 0.047).

#### Automated behaviour scoring

**Figure 4:**
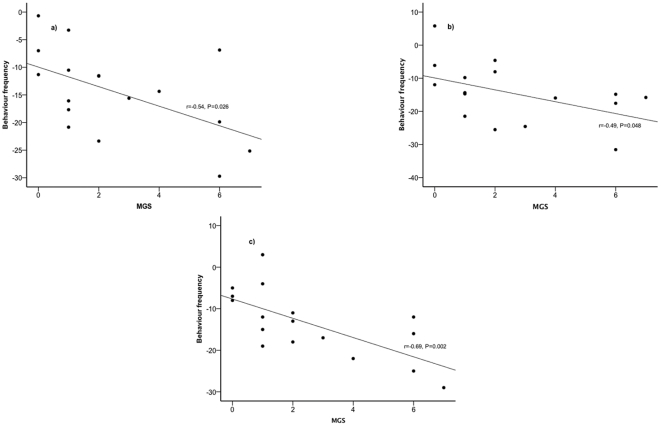
Relationship between changes in MGS and automatically scored behaviour. MGS scores of one experienced observer are presented on the x-axis and automatically scored behaviours from pre to post vasectomy are presented on the y-axis; walk left (a), walk right (b), and jump (c).

The change in MGS between the pre to post-operative period was negatively correlated with the change in three of the automatically scored behaviours: walk left (r = −0.57, P = 0.026), walk right (r = −0.49, P = 0.048), and jump (r = −0.69, P = 0.002) (see Figures 4a, b, c). The change in MGS was not correlated with the change in any of the remaining automatically scored behaviours (see Table 3).

**Table 3:** The correlation coefficients and P-values for the automated behaviours that were not significantly correlated with the MGS.

Behaviour	
Sniff (r = −0.342, P = 0.18)	Remain Partial Rear (r = −0.071, P = 0.79)
Stretch (r = 0.204, P = 0.43)	Come Down from Partial Rear (r = −0.161, P = 0.54)
Groom (r = 0.303, P = 0.18)	Rear Up from Partial Rear (r = −0.452, P = 0.07)
Pause (r = 0.361, P = 0.18)	Rear Up to Partial Rear (r = −0.182, P = 0.48)
Rear Up (r = −0.274, P = 0.28)	Repeat jumping (r = −0.380, P = 0.13)
Turn (r = −0.189, P = 0.47)	Remain Hang Cuddled (r = 0.087, P = 0.74)
Unknown (r = 0.02, P = 0.94)	Remain Hang Vertically (r = −0.190, P = 0.47)
Stationary (r = 0.230, P = 0.37)	Hang vertically from rear up (r = −0.338, P = 0.18)
Remain Low (r = 0.217, P = 0.40)	Remain Rear Up (r = −0.347, P = 0.17)
Walk Slowly (r = −0.269, P = 0.30)	Hang vertically from hang cuddled (r = −0.292, P = 0.26)
Come down (r = −0.272, P = 0.29)	Land vertically (r = −0.238, P = 0.36)
	Hang cuddled (r = −0.451, P = 0.08)

## Discussion

Facial expressions have long been considered as indicators of emotion in both human and non-human animals [11], and in humans they are routinely used to assess emotions such as pain, especially in those who are unable to communicate coherently (e.g. those with cognitive impairment and neonates [12]). Facial expressions in humans are reliably coded using the Facial Action Coding System (FACS), which describes the changes to the surface appearance of the face resulting from individual or combinations of muscle actions [13]. This anatomically based method has successfully been translated from human to non-human primate species, such as the chimpanzee (ChimpFACS [14]) and rhesus macaque (MaqFACS [15]), but has not been applied to assess pain in these species. The study by Langford and colleagues [9] represents the first successful attempt to assess pain via changes in facial expression in any animal species. Post-vasectomy changes in behaviour have been successfully assessed in various mouse strains using both manual and automated behavioural analysis [4], [5], [16].

In the present study, clear differences in Mouse Grimace Scale (MGS) were noted following a routine surgical procedure (scrotal approach vasectomy), with an increase in score from the pre to post-operative period. Analgesic treatment with either meloxicam or bupivacaine reduced the MGS score post-operatively compared to that observed in saline treated animals. The frequency of pain-related behaviours assessed using a manual scoring system (composite pain score) showed a similar pattern to that of the MGS and successfully differentiated between both the pre and post-operative periods, and the effects of analgesic treatment compared to control (saline). Those behaviours that have been previously shown to change in response to post-operative pain [4], [5], [16] were highly correlated with the MGS. The animals showing high MGS also exhibited high composite pain scores, high frequencies of wound licking and grooming and low frequencies of rearing. The correlation of manual behaviour analysis and the MGS and the ability of both techniques to successfully detect both changes from pre to post-vasectomy and the differences between the analgesic treatments make it likely that both are assessing the presence of post-surgical pain. The successful demonstration of this correlation between the two methods may well have been due to the still images scored using MGS being frame grabbed from the video used for the manual analysis. In other words, the same animals were scored by all methods at the same time period post-operatively.

The use of MGS for scoring post-operative pain has distinct advantages over that of the manual behaviour analysis, as manual analysis of behaviour is more complex because of a greater range of behaviours to potentially score. It is also more time-consuming to conduct (approximately 18 h compared to 1 h for complete scoring of 18 animals pre and post-operatively). Furthermore, changes in facial expressions of mice were detectable by relatively inexperienced observers with only the MGS manual for guidance. Manual behaviour analysis by comparison requires considerable training in order for observers to accurately and effectively score post-operative pain [17]. Finally, the assessment of pain using facial expressions in animals may have a further advantage over existing behavioural-based techniques, in that it capitalises on our potential natural tendency to focus on the face when interacting with animals. Leach et al. [10] demonstrated that when assessing pain in rabbits, even experienced observers focus predominately on the face rather than the body areas where behavioural indicators of pain are observed. This is not surprising, as humans have a tendency to focus on the face and in particular on the eyes of other people when attempting to assess emotions such as pain [18], [19].

Although the automated behavioural analysis was clearly able to detect behavioural changes from pre to post-vasectomy in mice, it was less successful at detecting analgesic effects post-operatively. This is most likely due to the current algorithm detecting primarily changes in normal activities such as walking, running and rearing, rather than changes in more pain-specific behaviours used in manual scoring. These changes in normal behaviour may result from not only post-operative pain but also from a more generalised stress response to surgery. Our previous studies have shown that anaesthesia in the absence of surgery has minimal effects in comparison to the effects of surgery [4]. It may be that the analgesic regimens used are successful in preventing some post-surgical pain, but are not completely effective, and so do not completely normalise behaviour. Alternatively, the abnormal pain-related behaviours that were influenced by analgesic treatment may reflect post-surgical pain, whereas the changes in normal activity may be part of a more generalized stress response. This is further supported by the relatively few correlations found between the automatically scored behaviours and the MGS, with the three negatively correlated behaviours (walk left and right and jumping) being directly related to general activity. Other studies using automated behavioural analysis have found similar reductions in general activity, but none of these have incorporated a wide range of analgesics, at varying dose rates [4], [5]. Clearly, a larger study including other types of surgery and other classes of analgesic, at varying dose rates, would enable a better evaluation as to which of these explanations is most likely.

Although a more extensive evaluation of the MGS is indicated, the present study suggests it will prove to be a quick and easy means of assessing post-surgical pain, and the efficacy of analgesic treatment in mice. Further, we consider that the sensitivity of the MGS can almost certainly be improved by obtaining higher resolution video under more optimal conditions that minimise artefacts such as reflections on the cage-front. Finally, it also seems likely that if facial expressions can be successfully applied in mice to assess pain, then it should also be appropriate for use in other animal species. This is supported by the recent development of the Rat Grimace Scale [20], which was developed using the same principle of Langford et al. [9] of assessing changes in facial expressions in rats undergoing routine rodent nociceptive models. These authors have also developed computer software (“Rodent Face Finder”) to automate the most labour intensive part of process; the locating of frames from video sequences in which the animal's face is clearly visible. This potentially increases the ease and speed with which facial expressions associated with post-surgical/procedural pain could be identified.

## Materials and Methods

### Ethical statement

All procedures were carried out under project and personal licences approved by the Secretary of State for the Home Office, under the United Kingdom's 1986 Animals (Scientific Procedures) Act and the Local Ethical Review Committee at Newcastle University. All the mice that were vasectomised in this study were required for use in the university's genetically modified mouse production programme. Consequently no animals underwent surgery or were directly used in order to record data for the purposes of this study. Verbal informed consent was gained from each participant prior to taking part in this study. Written consent was deemed unnecessary as no personal details of the participants were recorded. This study did not require institutional review board approval in order for it to be carried out. This study employed a strict ‘rescue’ analgesia policy. If any animal was deemed to be in greater then mild pain (assessed by an independent veterinarian), then buprenorphine (0.1 mg/kg sc) was immediately administered and the animal was removed from the study.

### Animals and husbandry

Eighteen male CD1 mice (Charles River Laboratories Inc, Margate, Kent, UK) weighing 30–40 g were used in this study. The mice were housed singly upon arrival in MB3 cages (30 cm×12 cm×12 cm: North Kent plastic cages Ltd, Kent, UK) for a 7-day acclimation period prior to the start of the study. During this time they were habituated to the general daily activity of the animal care staff, handling, weighing, the presence of the observer and the video monitoring equipment. The mice were housed singly throughout the study to enable video footage and still images to be obtained and to prevent changes in behaviour or facial expressions resulting from transient separation from their cage mates. The animal room was maintained at 22.5±1°C, 45% humidity and on 12/12 h light/dark cycle. Food (CRM (P), SDS Ltd, Essex, UK) and tap water were provided *ad libitum*. Sawdust bedding (Apsen, BS and S Ltd, Edinburgh, UK) was provided along with nesting material (Shredded paper, DBM, Broxburn, UK). A tunnel and nestlets were provided as environmental enrichment. The animals were free from any common pathogens in accordance with the FELASA health monitoring recommendations.

### Analgesic treatment groups

The mice were randomly assigned to one of three analgesic treatment groups. Group 1 (n = 6) acted as a control for analgesia and received a saline subcutaneous injection (2 ml/kg) administered 30 minutes prior to surgery. Group 2 (n = 6) received a subcutaneous injection of 20 mg/kg Meloxicam (Metacam: Boehringer Ingelheim, Labiana Life Sciences S.A. Terrassa, Spain) administered 30 minutes prior to surgery. Group 3 (n = 6) received 5 mg/kg of bupivacaine hydrochloride by local infiltration (Marcain 0.5%: AstraZeneca UK Ltd) into the wound site intra-operatively.

### Surgery

Thirty minutes prior to surgery the animals were weighed in order for the correct drug doses to be administered. Surgery began at 09:00 h with the same surgeon operating on all animals. Anaesthesia was induced with isoflurane in oxygen (Induction: 5% at 2 L/min, Maintenance: 2.5% at 0.5 L/min). All mice were placed onto bedding (VetBed, Kennel Needs and Feeds, Morpeth, UK) with a heat mat (Harvard apparatus, Edenbridge, Kent, UK) underneath to maintain body temperature. The scrotum was shaved and then cleaned with chlorhexidine (Hydrex Dermaspray, Adams Healthcare, Leeds UK). Surgery involved a 1 cm longditudinal incision made through the skin and scrotum wall. The vas deferentia were located and a small piece removed using cautery. The incision in the tunica vaginalis was closed with Vicryl 5.0 (Johnson & Johnson, Belgium). Tissue glue (Nexaband, Abbott laboratories, Chicago) and sutures (Vicryl 5.0) were used to close the skin. Anaesthesia lasted 10±2 min, following, which the mice recovered in an incubator, maintained at 35±1°C for 30 min. They were then returned to their home cages and transferred to a quiet room for filming. No intra-operative complications were reported and all mice recovered from anaesthesia uneventfully.

### Video Recording

On the day prior to surgery and one hour after surgery, the mice were placed individually in clear ‘1284’cages (35 cm×20 cm×14 cm) (Techniplast UK Ltd) which contained only bedding (Aspen). The mice were allowed to acclimate for 10 minutes. The rear and one side wall of the cage were made opaque in order to reduce any reflections during filming. The mice were filmed for 12 minutes using two High Definition Cameras (Sony High Definition HandyCam model HDR-XR155, Sony, Japan). The cameras were placed at fixed distance from the cage, with one on the short side and one on the long side of the cage. This setup gave the highest probability of capturing the faces of the mice during filming. Following filming, all mice were returned to their home cages. Following the post-operative filming each mouse also received a subcutaneous injection of 0.05 mg/kg buprenorphine (Vetergesic; Alstoe Animal Health) to ensure they received effective analgesia after their initial assessment.

### Mouse Grimace Scale (MGS)

From each 10 minute video sequence still images (frame-grabbing) were taken whenever the mouse was found to be directly facing the camera, enabling generation of a number of clear and high quality images of each mouse pre and post-vasectomy. Each image was cropped so that only the head and not the body of the mouse were visible. This prevented the observers from being biased by the body of the animal when attempting to score facial expressions [9]. From these images, sixty were selected at random by a non-participating assistant for further scoring and comprised 30 pre and 30 post vasectomy images. The 30 post-vasectomy images were comprised of 10 images of mice from each treatment group. This image set comprised between 1 and 2 images of each mouse pre and post vasectomy. An individual who was experienced in the use of the scoring system scored this image set. This individual's scores were considered the most accurate, and used to compare with the behavioural analysis data. Twenty observers who were given only minimal training in use of the system also undertook scoring. The scores from these 20 observers were used to assess the ability of MGS scoring to detect the effects of pain and analgesic treatment.

The sixty images were scored in a random order using the Mouse Grimace Scale [9] by the treatment and session (pre or post vasectomy) blind participants. Briefly, each participant was given a description and a pictorial guide (see Figure 1: Langford et al. [9]) of each of the five Facial Action Units (FAUs) that comprise the MGS; orbital tightening, nose bulge, cheek bulge, ear position and whisker position (Please see Langford et al. [9] for a detailed description of these FAUs). They were then asked for each image to give a score for each of FAU using a 3-point scale (0 = not present, 1 = moderately present & 2 = obviously present). If the participant was unable to see a particular FAU clearly, they were asked not to score it and to state that they could not determine it.

### Participant selection

A total of 20 observers participated in this study and were recruited and tested in 2010 at Newcastle University. The observers were from diverse backgrounds and included veterinary surgeons, veterinary nurses, research scientists, animal technicians, psychology students and non-animal related occupations.

### Behavioural Scoring

The behaviour observed in each video sequence (10 min epoch) was scored using both manual and automated behavioural scoring. Manual scoring was carried out by one treatment-blind observer using Observer XT (Version 8: Noldus Information Technology, Wageningen, Netherlands) according to an ethogram developed for assessing post vasectomy pain in mice (see Table 4). Automated behavioural scoring was carried out using HomeCageScan (Version 3, CleverSystems Inc., Reston, USA) according to a pre-programmed ethogram of general mouse behaviours (see Table 5). The same 10 min epoch was scored using both methods. The Observer XT and HomeCageScan software was used to calculate the frequency and duration of the behaviours that were recorded.

**Table 4:** Ethogram for manual behavioural analysis (adapted from Miller et al. [5]).

Behaviour	Description
Arch	Arching of back
Dig	Digging into the bedding
Flinch	Small movement involving whole body
Groom	Grooming
Hop	Hopping movement
Jump	Jumping
Lie	Lying down
Press	Pressing abdomen towards cage floor
Rear	Standing on rear legs
Rear Leg Lift	Lifting of one of the rear legs
Scratch	Scratching
Sit	Partial crouch, weight resting on hind limbs
Sniff	Sniffing
Stagger	Partial loss of balance when walking
Stand	Inactive
Swim	Swimming movement through cage bedding
Turn	Change in direction mouse is facing
Twitch	Rapid contraction of back muscles
Unknown	Undefined behaviour
Walk	Walking
Wobble	Slight side to side movement
Wound Lick	Licking of the surgical wound
Writhe	Contortion of abdominal muscles

**Table 5:** Ethogram for automated behavioural analysis (adapted from Miller et al. [5]).

Behaviour	Description
Circle	Movement in a circular motion
Come Down from Rear Up	Coming down from a reared position
Come Down from Partial Rear	Coming down from a partial rear position
Groom	Grooming
Jump	Jumping
No Data	HomeCageScan failed to collect data
Pause	Period of no movement
Partial Rear	Crouching on rear legs supported or unsupported
Rear Up	Standing on rear legs supported or unsupported
Rear Up from Partial Rear	Moving from partial to full rear
Remain Hang Cuddled	Hanging in a cuddled posture
Remain Hang Vertically	Hanging with a vertical posture
Remain Low	Remaining low to cage floor
Remain Partial Rear	Duration in partial rear position
Remain Rear Up	Duration in the reared position
Sleep	Sleeping
Sniff	Sniffing
Stationary	Inactive/not moving around the cage
Stretch Body	Full body stretch
Turn	Change in direction mouse is facing
Twitch	Rapid localised movement of the body
Unknown	Behaviour not recognised by HomeCageScan
Walk Left	Normal walking speed to the left
Walk Right	Normal walking speed to the right
Walk Slowly	Walking slower than normal in either direction

### Data analysis

The mouse grimace scale was determined using a slight modification of the method developed by Langford et al. [9]. In this study the MGS was a composite of the FAU's; orbital tightening, nose bulge, cheek bulge and ear position but not whisker position. The majority of our participants were unable to score whisker position in many of the images, as they were not of high enough quality for whisker position to be clearly seen. Consequently, we chose to exclude whisker position prior to any analysis. In order to explore the effect of time (pre vs. post vasectomy) and analgesic treatment, the mean MGS scores were calculated for each image pre (n = 30) and post vasectomy (n = 30) across all twenty participants. The MGS scores of a single participant (MCL) with experience of scoring the mouse FAU's were used to explore the relationship between changes in behaviour and MGS observed from the pre to post-vasectomy periods. In order to investigate this relationship the change in MGS score was calculated using a single pre and a single post vasectomy image for each of the 18 mice that were randomly selected. This was compared with the change in frequency of manually and automatically scored behaviours for the same mice.

### Statistical Analysis

All statistical analyses were conducted using SPSS 18 (SPSS Inc., Chicago, USA). The data were normally distributed with homogeneity of variance, so parametric analyses were carried out. Differences were considered to be statistically significant if P<0.05. Repeated measures analysis of variance was used to analyse the data with the time points (pre and post-vasectomy) as the within-subjects factor and the treatment group as the between-subjects factor. Any time*treatment interactions were further investigated using multivariate analysis of variance with data from the separate time periods forming the dependent variables and treatment group as the between subjects factor. Post-hoc analysis of treatment group effects was conducted using Bonferroni post-hoc test. Pearson correlation coefficients were calculated to investigate the relationship between the changes in behaviour and MGS observed from pre to post-vasectomy.
